# Feasibility of an antegrade-retrograde single-sheath inverse technique via vertical puncture in dysfunctional hemodialysis arteriovenous fistula angioplasty

**DOI:** 10.1186/s42155-024-00480-4

**Published:** 2024-09-20

**Authors:** Tetsuya Hasegawa, Masahiro Tsuboi, Yuki Takahashi, Akira Endo, Yasuo Gotoh

**Affiliations:** 1 Department of Diagnostic Radiology, Japanese Red Cross Ishinomaki Hospital, 71, Nishimichishita, Hebita, Ishinomaki-shi, Miyagi 986-8522 Japan; 2 Department of Diagnostic Radiology, Osaki Citizen Hospital, 3-8-1, Honami, Furukawa, Osaki-shi, Miyagi 989-6183 Japan; 3 Department of Radiology, Shunt Clinic Sendai-Higashi, 2-17-25, Higashi Sendai, Miyagino-ku, Sendai-shi, Miyagi 983-0833 Japan

**Keywords:** PTA, Sheath, Inverse technique, Arteriovenous fistula, Dialysis access, Vascular access

## Abstract

**Background:**

Stenosis resulting in dysfunctional dialysis access may occur simultaneously on the anastomotic and central venous side. The purpose of this study was to retrospectively evaluate the feasibility of a single sheath inverse technique using the vertical puncture approach to perform bidirectional transvenous percutaneous transluminal angioplasty (PTA) from a single sheath for such dialysis access stenoses.

**Materials and Methods:**

Twenty patients (26 cases; 13 males; median age, 74 [range: 50–89] years) who underwent PTA using the sheath inverse technique for dysfunctional arteriovenous fistula stenoses between April 2019 and June 2023 were included. All procedures were performed in an outpatient setting. A 4-cm sheath (4Fr, four cases; 5Fr, 19 cases; 6Fr, three cases) was inserted by vertical puncture through a cutaneous vein in the forearm (20 cases) or upper arm (six cases). After treating one side of the lesion, the sheath was reversed to treat the lesion on the opposite side. The vessel diameter at the sheath insertion site, the success rate of sheath inversion, the number of PTA balloon catheters used, the PTA success rate, adverse events, and primary and secondary patency rates up to one year after PTA were evaluated.

**Results:**

The median diameter at the sheath indwelling site was 5.2 (range: 3.6–9.5) mm, and sheath inversion was successful in all cases, eliminating the need to place an additional sheath at another site for contralateral stricture treatment. The number of balloon catheters used was one and two in 17 (65%) and eight cases (31%), respectively, and three in one case wherein a drug-coated balloon was used. PTA was successful in all cases and major complications were not observed. However, in one case wherein a sheath had to be placed at the arterial needle puncture site, the skin was hard, leading to difficulty in inversion, and transient venous spasm occurred post-inversion. The primary patency rates at 3, 6 and 12 months after the PTA were 87.5%, 41.7%, and 20.8%, respectively. The secondary patency rates at 6 and 12 months were 100% and 75%, respectively.

**Conclusion:**

The single-sheath inverse technique for arteriovenous fistulas was feasible without sheath withdrawal.

**Graphical Abstract:**

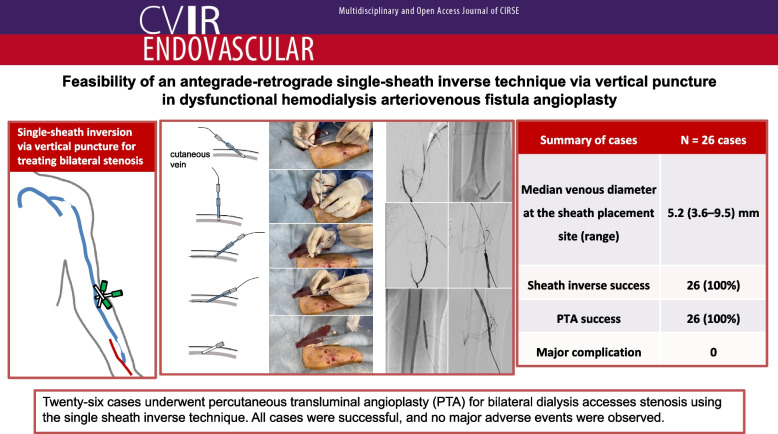

**Supplementary Information:**

The online version contains supplementary material available at 10.1186/s42155-024-00480-4.

## Background

Percutaneous transluminal angioplasty (PTA) remains the first-line treatment for dialysis access stenosis. It is indicated when there is significant stenosis accompanied by clinical dysfunction. Clinical signs of access dysfunction are broadly manifested as inflow obstruction (difficulty in puncturing, inability to achieve adequate dialysis flow rate, and poor fistula maturation) or outflow obstruction (swelling of arm and prolonged bleeding) [1, 2]. The transvenous retrograde approach is commonly used in PTA for stenosis of the arteriovenous fistula (AVF) for dialysis, as most patients have stenosis on the anastomotic side [3–6]. However, when lesions are present both upstream (anastomosis side) and downstream (heart side) of the approach site, the two sheaths may need to be inserted in opposite directions. According to the Japanese Society of Interventional Radiology guidelines for basic techniques of vascular access interventional therapy, “In cases of difficulty to insert two sheaths, it can be possible that one sheath is inverted oppositely using a guidewire” [7]. However, to the best of our knowledge, this detailed, single-sheath inverse technique has rarely been reported in the literature. Therefore, in this study, we aimed to retrospectively evaluate the feasibility of the single-sheath inverse technique using a vertical puncture approach in PTA for AVF stenosis for dialysis.

## Methods

This is a retrospective observational study conducted at a single institution. All procedures were performed in accordance with the ethical standards of our institutional research committee. The study was approved by our Institutional Review Board (approval number: 20240325–53), and all patients provided written informed consent to undergo the procedure.

PTA procedures for AVF stenosis and occlusion are typically performed on an outpatient basis. Therefore, the transvenous approach is prioritized. The puncture site is chosen by the operator based on the most feasible location, determined through pre-procedural ultrasonography. Specifically, sites with a longer intact section and where the vessel diameter is relatively preserved are selected. If the examination, ultrasound findings, and dialysis indicators (such as increased venous pressure or upper limb edema) indicate stenoses on both sides of the sheath insertion site or if stenosis is suspected, the sheath may be reversed. Therefore, the puncture is performed vertically.

### Patients

Between April 2019 and June 2023, 20 patients (13 males and 7 females; median age [range], 74 [50–89] years) underwent 26 PTA procedures for AVF stenosis using the single-sheath inverse technique (Table 1). The median number of past PTA performed until this intervention was 3 (range, 1–9). All patients were referred to our outpatient department and had reduced blood flow for dialysis, as well as at least one of the following symptoms: upper limb edema, prolonged hemostasis time, or increased venous pressure. Patients with arteriovenous grafts were excluded. In cases of thrombosed occluded AVF, there were no cases with stenosis downstream (toward the heart) from the sheath insertion site; thus coincidentally, they were not included in this study. Before inserting the sheath, ultrasonography was performed to observe stenosis on the anastomotic side. The properties of the sheath insertion site, including its diameter, were evaluated in all but two cases. The sheath insertion sites were the cephalic vein of the forearm in 19 cases (73%), cephalic vein of the upper arm in six cases (23%), and ulnar vein of the forearm in one case (4%) of ulnobasilic AVF.

**Table 1:** Details of PTA using the single-sheath inverse technique in 26 cases

**Patient Characteristics**	***N* = 20 patients**	
Median age (range)	74 (50–89) y	
Sex (Male: Female)	13: 7	65%: 35%
Median number of past PTA (range)	3 (1–9) times	
AVF type		
Radiocephalic AVF	15	75%
Brachiocephalic AVF	4	20%
Ulnobasilic AVF	1	5%
**Summary of cases**	***N* = 26 cases**	
Sheath insertion site		
Cephalic vein of the forearm	19	73%
Cephalic vein of the upper arm	6	23%
Ulnar vein of the forearm	1	4%
Size of sheath used		
4 Fr	4	15%
5 Fr	19	73%
6 Fr	3	12%
Median venous diameter at the sheath placement site (range)	5.2 (3.6–9.5) mm	
Sheath inverse success	26	100%
Number of balloon catheters used		
One	17	65%
Two	8	31%
Three	1^a^	4%
PTA success	26	100%
Major complication	0	
Minor complication	1^b^	4%
Primary and secondary patency rates in 24 cases	(primary; secondary)	
3 months	87.5%; 100%	
6 months	41.7%; 100%	
1 year	20.8%; 75%^c^	

*AVF* arteriovenous fistula

^a^Use of drug-coated balloons

^b^Transient venous spasm occurred

^c^surgical reconstruction: 3, death: 2, tunneled cuffed catheter: 1

### Procedures

**Fig. 1:**
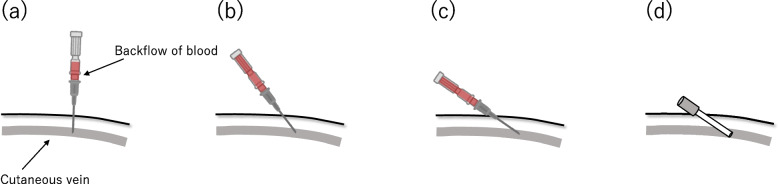
Schema of the vertical puncture approach. **a** First, the skin and cutaneous vein were perpendicularly punctured using an 18-gauge needle. **b**, **c** After confirming the backflow of blood into the inner hub, the needle was tilted to one side. **d** Finally, a suitably sized sheath was inserted using the Seldginger technique

A 4-cm sheath (Radifocus introducer; Terumo Co., Tokyo, Japan) was inserted in 26 cases (4Fr, *n* = 4; 5Fr, *n* = 19; 6Fr, *n* = 3) using the vertical puncture approach with the Seldinger technique. The vertical puncture approach involved perpendicularly puncturing the skin with an 18G needle; after confirming the backflow of blood into the inner hub, the needle was tilted to one side (Fig. 1). First, a sheath was inserted toward one side, and balloon angioplasty was performed for the stenosis. Next, the sheath was reversed to treat the lesion on the opposite side using the same or a different balloon catheter. Balloon catheters with diameters ranging from 3 to 8 mm were used. In one case, after sheath inversion, the sheath was changed to 6Fr to allow the use of a larger balloon. There are two main methods for inverting the sheath using a dilator and a guidewire attached to the sheath introducer set: with and without fluoroscopy.

**Fig. 2:**
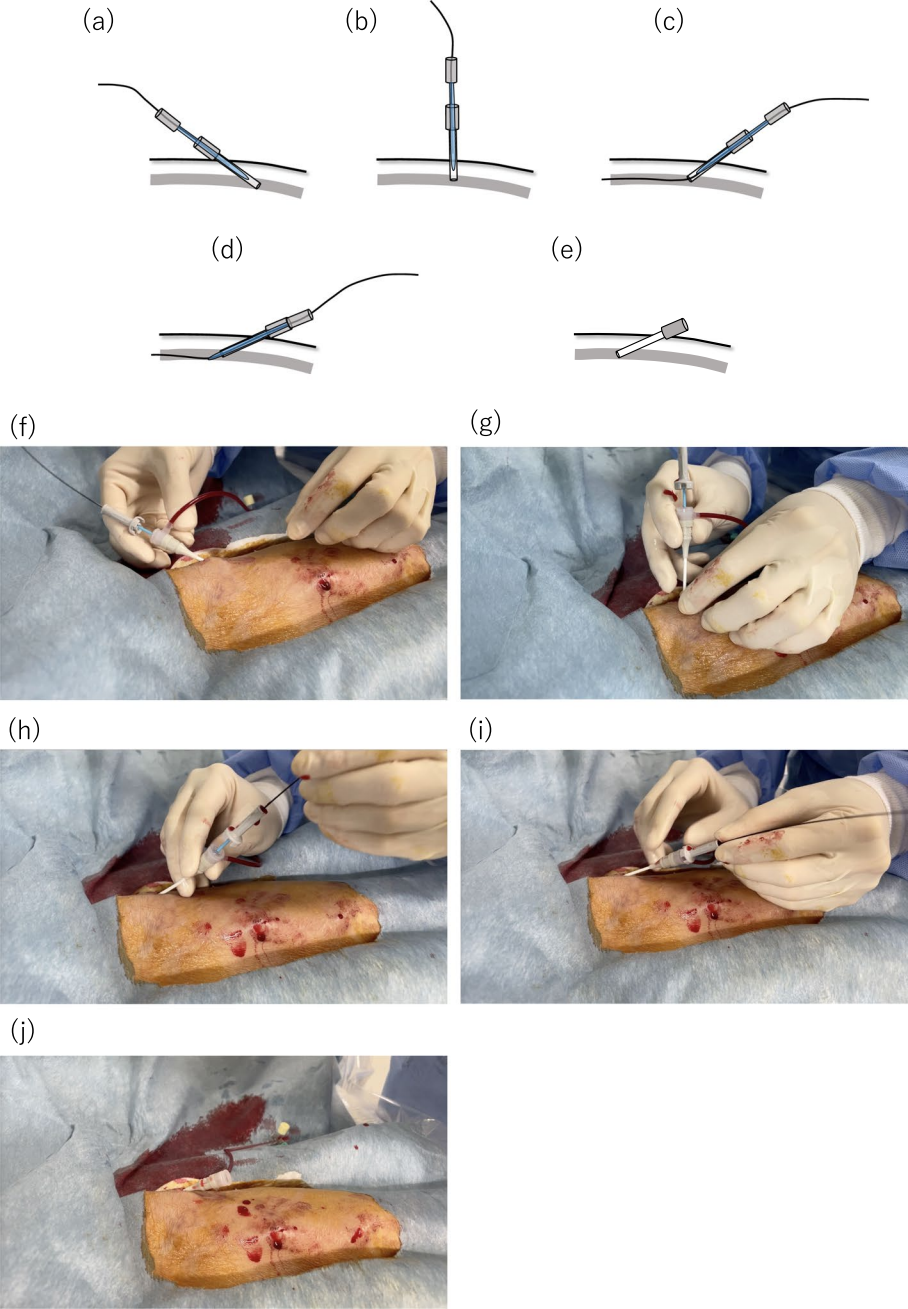
Schematic and sequential images demonstrating the sheath inverse technique without fluoroscopy. **a**-**f** First, the dilator and guidewire were loaded into the sheath to ensure that they remained within the sheath's tip, preventing kinking of the sheath and facilitating the rapid use of the guidewire post-inversion. **b**-**g** The sheath was then carefully withdrawn in a vertical direction along with the dilator and guidewire, encountering some resistance. **c**-**h** The sheath was slightly inversed, and the guidewire advanced to the opposite side, ensuring smooth progression without resistance. **d**-**i** The dilator was advanced to emerge from the sheath’s tip, and the sheath was then advanced with the dilator over the guidewire. **e**-**j** Upon adequate sheath insertion, the guidewire and dilator were removed

**Fig. 3:**
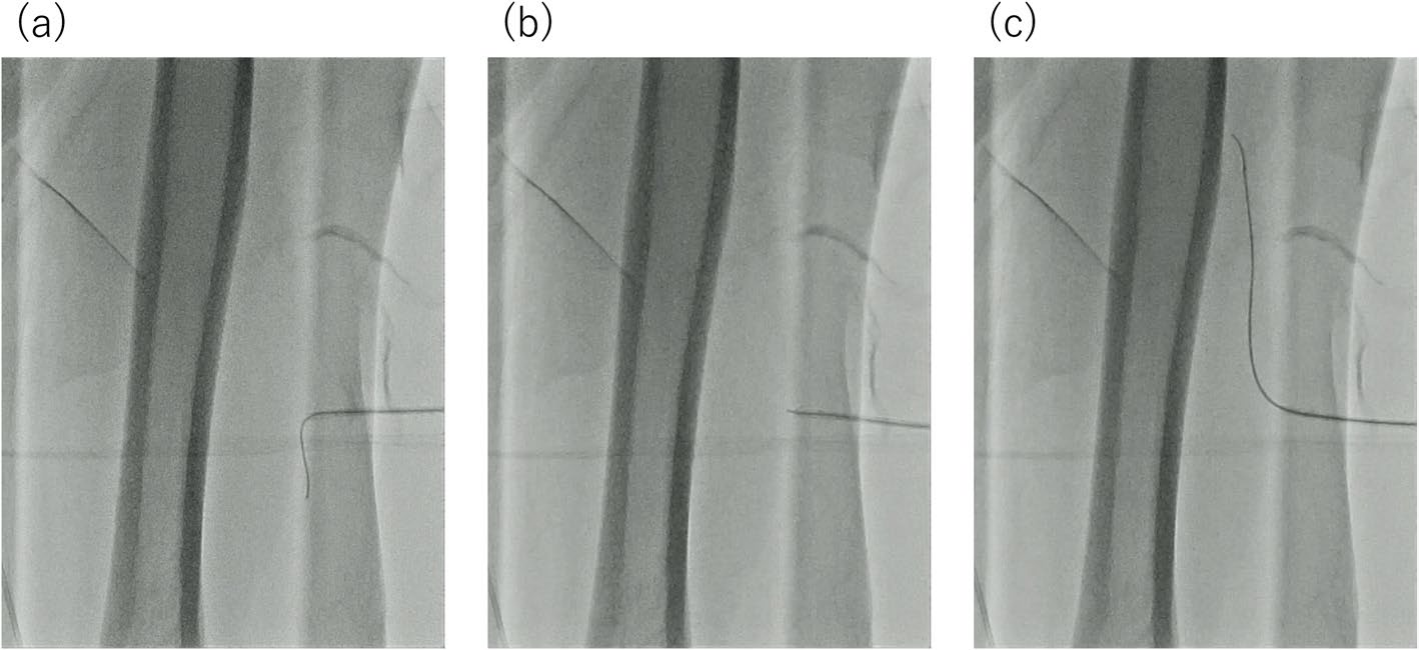
Fluoroscopy-assisted demonstration of the sheath inverse technique. **a** The dilator was loaded into the sheath to prevent it from protruding beyond the tip; this was followed by pulling out the sheath under fluoroscopy while maintaining visualization of the blood vessel with the guidewire. **b**, **c** The opposite side was selected using the guidewire while ensuring the sheath was in a near-vertical position. Upon sufficient advancement of the guidewire, we proceeded to advance and position the dilator and sheath accordingly

**Fig. 4:**
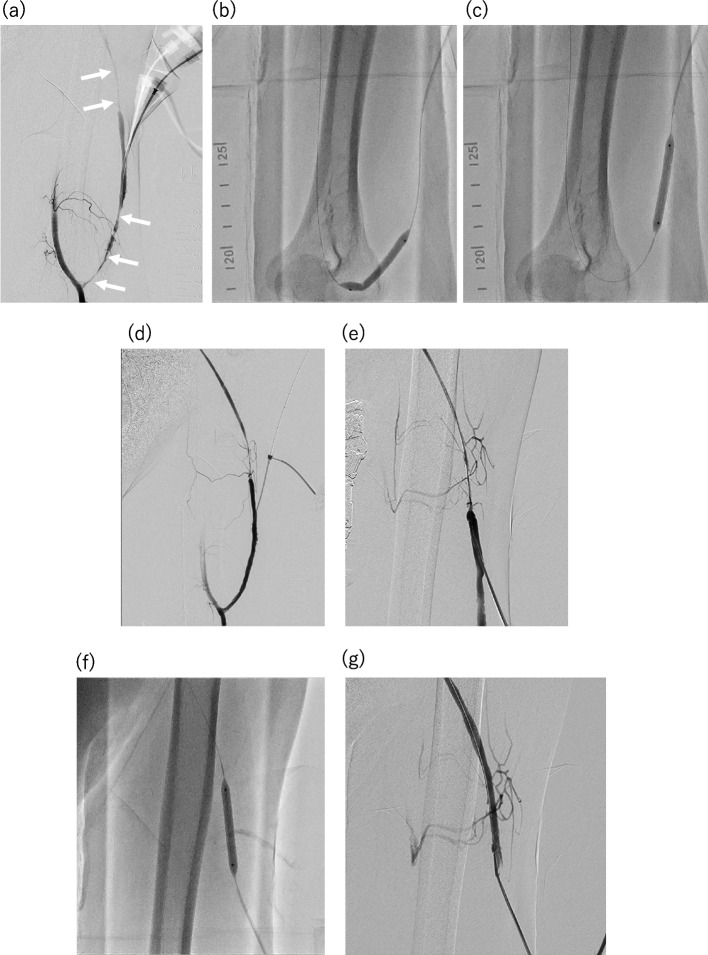
Case presentation of a male patient in his 50 s with a left brachiocephalic arteriovenous fistula (AVF). The patient presented with complaints of low blood flow quantity and prolonged hemostasis time at the arterial needle puncture site. The vessel diameter at the sheath insertion site was φ3.6 mm. **a** A 4Fr sheath was placed facing the anastomotic side, followed by antegrade angiography from the brachial artery using a 4Fr short catheter to confirm severe stenoses at the anastomotic side and the opposite side of the sheath (indicated by arrows). **b c** Dilation of the severe stenosis on the anastomotic side using a 5 mm × 40 mm high-pressure balloon catheter (BRAVUS, Boston Scientific, Natick, Massachusetts). **d** Retrograde angiography from the sheath confirmed the resolution of the severe stenosis on the anastomotic side. **e** Confirmation of residual stenosis on the opposite side after sheath inverse technique through antegrade angiography. **f** Dilation of the residual stenosis using the same balloon catheter. **g** Verification of the resolution of the severe stenosis

- Without fluoroscopy: The dilator and the short guidewire provided with the sheath introducer set are loaded into the sheath and allowed to remain within the sheath’s tip to prevent the sheath from kinking. As the sheath tip slightly traces the posterior wall of the vessel with minimal resistance, the sheath is slowly retracted and erected. When the sheath stands vertically, the guidewire is advanced into the opposite vein. Subsequently, the dilator is advanced from the tip of the sheath, followed by advancing the sheath over the guidewire (Fig. 2).

b) Under fluoroscopy*: *Similar to method (a), the dilator is loaded into the sheath tip to prevent sheath kinking. In this method, the 0.035” short guidewire attached to the sheath introducer is set slightly protruding from the sheath tip. Then, the sheath is pulled slowly to the vertical position, and traced along the posterior wall of the vessel with slight resistance under fluoroscopy. The guidewire is sufficiently advanced deep into the opposite vein, and its progression is confirmed with fluoroscopy. Subsequently, the dilator is advanced from the tip of the sheath, followed by the sheath itself, as described in method (a) (Figs 3 and 4).

### Outcome points

The evaluation items were the vessel diameter at the sheath insertion site, the success rate of the sheath inverse technique, the number of PTA balloon catheters used, the PTA success rate, and adverse events. In addition, primary and secondary patency rates at 3 months, 6 months, and one year after the PTA were evaluated. The diameters of the blood vessels at the sheath insertion site were measured using ultrasonography without using a tourniquet. Successful sheath inverse was defined as reversal without sheath withdrawal. PTA success was defined as technical residual stenosis of < 30% and the clinical ability to provide at least one session of adequate dialysis after percutaneous intervention [8, 9]. Adverse events were evaluated using the CIRSE classification system [10]. After excluding two cases lost to follow-up immediately after PTA, the patency rates were evaluated in 24 cases. Primary patency was defined as patency during the interval between the PTA using single-sheath inverse technique and the next repeated radiologic intervention for dysfunction in dialysis access. Secondary patency was defined as patency during the interval between the PTA and the occurrence of any of the following events: surgical revision or reconstruction of the fistula, tunneled cuffed catheter placement, or death. Multiple treatments including angioplasty and thrombectomy may be included in secondary patency [11].

## Results

**Fig. 5:**
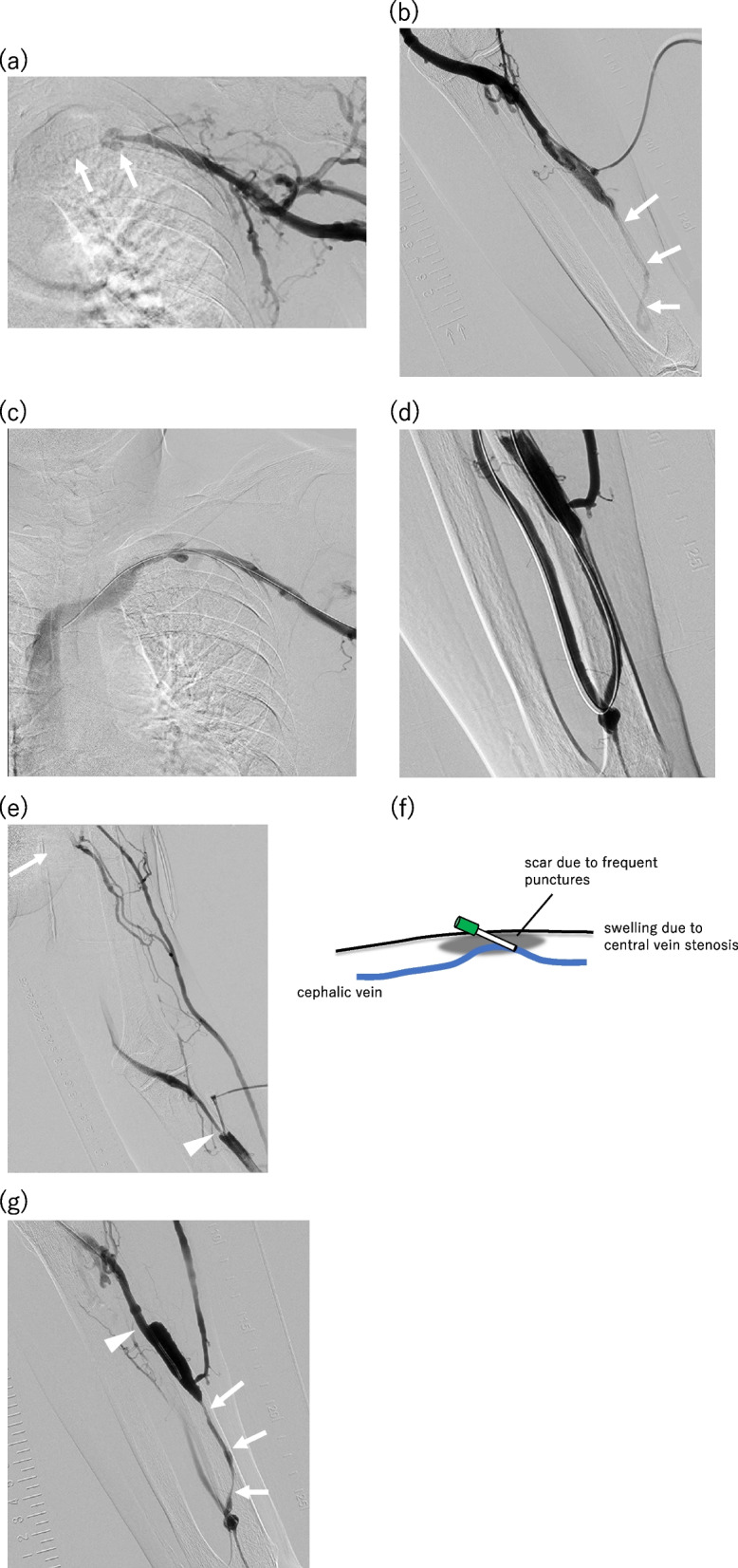
Case study of a male patient in his 70 s with a left radiocephalic arteriovenous fistula (AVF). The patient presented with swelling of the vascular access limb and low blood flow quantity. The vessel diameter at the sheath insertion site was φ4.7 mm. **a** Placement of a 5Fr sheath facing the heart side, followed by antegrade angiography revealing severe stenosis of the subclavian vein (indicated by arrows). **b** Prior to the sheath inverse technique, retrograde angiography demonstrated slight visualization of the stenosis on the anastomotic side (indicated by arrows) using a tourniquet. **c** First, the subclavian venous stenosis was dilated using a Φ6 mm × 40 mm balloon catheter (Ultraverse035; Becton Dickinson, Franklin Lakes, NJ, USA), resulting in stenosis reduction. **d** After sheath inversion, the stenosis of the forearm cephalic vein was dilated using a Φ5 mm × 40 mm balloon catheter (DORADO; Becton Dickinson), resolving the issue. **e** However, a transient spasm—presumed to be a result of the sheath inverse technique—occurred at the site of inversion (indicated by arrowhead), where no stenosis was observed before inversion. **f** The cephalic vein ran deeply throughout the patient, with swelling of the AVF limb due to downstream venous (central vein) stenosis. The arterial needle puncture site was the only easily accessible site, which had scars formed by frequent dialysis punctures. **g** The next PTA was performed 182 days later for treatment of insufficient blood flow. Severe restenosis was observed in the cephalic vein near the anastomosis before balloon dilatation image (arrows). There was no stenosis in the spasm site of the previous sheath reversal (arrowhead)

The median venous diameter at the sheath placement site was 5.2 (range: 3.6–9.5) mm. Sheath inverse was successful in all 26 cases. The number of balloon catheters was one in 17 cases (65%), and two in eight cases (31%). Three balloons were used in one case (4%) using a drug-coated balloon catheter. PTA was successful in all cases. In all cases, we confirmed a reduction in the manual increase in venous pressure from the sheath after PTA, and there was no difficulty in achieving hemostasis at the sheath placement site. Although no major complications were observed, there was one case of temporary venous spasm after the sheath inverse technique. In this case, the sheath needed to be placed at the arterial needle puncture site; however, the skin at this site was hard due to the presence of scar tissue, leading to difficulty in inversion (Fig. 5). The primary patency rates at 3 months, 6 months and 1 year after the PTA were 87.5% (21/24), 41.7% (10/24), and 20.8% (5/24), respectively. The secondary patency rates at 3 months, 6 months and 1 year after the PTA were 100%, 100%, and 75% (18/24), respectively. The results are summarized in Table 1, while case details including clinical course post-PTA are shown in Supplemental Table 1.

## Discussion

Juxta anastomosis, defined as the initial 5 cm of the AVF starting at the arterial-venous anastomosis, is the most common location of stenosis in the venous segments in native AVF for dialysis [3–6, 12, 13]. In contrast, the central vein consists of the subclavian vein, brachiocephalic vein (previously called the innominate vein), or superior vena cava, and the cephalic arch can cause AVF stenosis [3, 5, 12, 14–16]. Although rare, both sites may be stenosed [4, 17]. Dilating both stenoses usually requires placing two sheaths in opposite directions or performing an anterograde angioplasty via brachial arterial access [18].

The single-sheath inverse technique is occasionally used by experienced PTA operators for hemodialysis access trouble, whether AVF or arteriovenous graft. The sheath inverse technique is not difficult to perform. However, there are only two case reports of the sheath inverse technique [17, 19], and only one case of PTA for hemodialysis access [17]. Takashima et al. [17] stated that the advantages are “pain reduction, shortened operation time, and cost-savings.” However, there are no detailed descriptions of these technical aspects. In this study, sheath inverse was possible without the withdrawal of a median vessel diameter of approximately 5 mm.

If a sheath were placed from the radial artery, all stenoses could be eliminated by antegrade access with a single sheath without sheath inversion. However, because PTA is performed on an outpatient basis, transvenous access is preferred to reduce the risk of complications such as hematoma at the puncture site. In addition, it is difficult to place a 6Fr sheath for transarterial access on an outpatient basis safely.

However, one patient experienced temporary venous spasms, which may have been due to sheath inverse. In this case, the cephalic vein was generally deep throughout the body. In addition, the upper limb on the hemodialysis access side was swollen due to severe stenosis of the subclavian vein. Therefore, the arterial needle puncture site was the only easy puncture site. However, the inversion of the sheath was not smooth owing to scarring caused by frequent punctures for dialysis (Fig. 5). In areas with skin hardness, such as scarring, it may be better to avoid sheath inverse.

According to a recent review article by Ratnam et al. [2], primary patency of the target lesion at 6 and 12 months was 42–63% and 23–50.5%, respectively [3, 20–22]. In the present study, the primary patency rate was 41.7% at 6 months and 20.8% at 12 months, which are not extremely low compared to those of previous reports, even though our cases had a history of multiple PTAs and conditions were not favorable. We believe this indicates that the sheath reversal method does not cause excessively poor patency rates.

Limitations include the fact that this was a retrospective study with a small number of patients at a single institution; additionally, the depth of the blood vessel at the sheath insertion site and length of the intact portion were not evaluated, and the skin firmness was not rated.

In conclusion, a sheath-inverse PTA technique for AVF for hemodialysis access is feasible without withdrawal.

## Supplementary Information

Supplementary Material 1.

## Data Availability

The datasets used and analyzed during the current study are available from the corresponding author on request.

## Abbreviations

AVF: Arteriovenous fistula
PTA: Percutaneous transluminal angioplasty
IRB: Institutional Review Board
