# Supplementary material for: Feasibility of an antegrade-retrograde single-sheath inverse technique via vertical puncture in dysfunctional hemodialysis arteriovenous fistula angioplasty
Source: CVIR Endovasc. 2024 Sep 20;7:69. doi: 10.1186/s42155-024-00480-4 (PMC11415322; doi:10.1186/s42155-024-00480-4)
Supplement: Supplementary file 1 — Supplementary Material 1. [file 42155_2024_480_MOESM1_ESM.docx]

**Supplemental Table 1. Case details including clinical course of post PTA**

| Case | AVF type | AVF trouble for central side | access site | Diameter of  access site (mm) | Sheath size  (Fr.) | Complication | 3 months | 6 months | 12 months |
| --- | --- | --- | --- | --- | --- | --- | --- | --- | --- |
| 1 | Rt. RCAVF | elevated VP | Cephalic vein of forearm | 4.9 | 4 |  | pp | PTA | death |
| 2 | Rt. RCAVF | elevated VP | Cephalic vein of forearm | 6.6 | 5 |  | pp | pp | PTA |
| 3 | Lt. RCAVF | elevated VP | Cephalic vein of forearm | 7.3 | 5 |  | pp | PTA | reconstruction |
| 4 | Rt. RCAVF | elevated VP | Cephalic vein of upper arm | 7.9 | 5 |  | lost follow-up | − | − |
| 5 | Lt. RCAVF | elevated VP | Cephalic vein of forearm | − | 4 |  | pp | pp | pp |
| 6 | Rt. RCAVF | elevated VP | Cephalic vein of forearm | 4.8 | 5 |  | pp | pp | pp |
| 7(1) | Rt. RCAVF | elevated VP | Cephalic vein of forearm | − | 5 |  | pp | PTA | sp |
| 8(1) | Rt. RCAVF | severe stenosis on US | Cephalic vein of upper arm | 5.8 | 5 |  | pp | pp | pp |
| 7(2) | Rt. RCAVF | elevated VP | Cephalic vein of forearm | 5.3 | 5 |  | pp | pp | PTA |
| 9 (Fig. 4) | Lt. BCAVF | severe stenosis on US | Cephalic vein of upper arm | 3.6 | 4 |  | pp | PTA | sp |
| 10 | Lt. BCAVF | prolonged hemostasis time | Cephalic vein of upper arm | 3.8 | 4 |  | PTA | sp | reconstruction |
| 11(1) | Lt. RCAVF | prolonged hemostasis time, elevated VP | Cephalic vein of forearm | 6.5 | 5 |  | pp | PTA | sp |
| 12 | Lt. BCAVF | elevated VP | Cephalic vein of upper arm | 7.1 | 5 |  | pp | pp | pp |
| 7(3) | Rt. RCAVF | elevated VP | Cephalic vein of forearm | 5 | 5 to 6 |  | pp | pp | PTA |
| 11(2) | Lt. RCAVF | prolonged hemostasis time, elevated VP | Cephalic vein of forearm | 4.3 | 5 |  | pp | PTA | sp |
| 13 | Rt. RCAVF | severe stenosis on US | Cephalic vein of forearm | 4.5 | 6 |  | pp | PTA | sp |
| 11(3) | Lt. RCAVF | severe stenosis on US | Cephalic vein of forearm | 6.3 | 5 |  | pp | PTA | sp |
| 14 | Rt. BCAVF | elevated VP | Cephalic vein of upper arm | 4.4 | 6 |  | lost follow-up | − | − |
| 15(1) | Rt. RCAVF | severe stenosis on US | Cephalic vein of forearm | 4 | 5 |  | PTA | sp | sp |
| 16  (Fig. 5) | Lt. RCAVF | upper limb edema | Cephalic vein of forearm | 4.7 | 5 | venous spasm | pp | pp | PTA |
| 17 | Lt. RCAVF | prolonged hemostasis time | Cephalic vein of forearm | 4.3 | 5 |  | pp | PTA | sp |
| 18 | Rt. UBAVF | prolonged hemostasis time | Ulnar vein of forearm | 5.9 | 5 |  | PTA | sp | sp |
| 8(2) | Rt. RCAVF | severe stenosis on US | Cephalic vein of forearm | 7.7 | 6 |  | pp | PTA | TCC |
| 19 | Rt. RCAVF | severe stenosis on US | Cephalic vein of forearm | 5.7 | 5 |  | pp | pp | pp |
| 15(2) | Lt. RCAVF | elevated VP | Cephalic vein of forearm | 4.7 | 5 |  | pp | PTA | reconstruction |
| 20 | Rt. RCAVF | elevated VP | Cephalic vein of forearm | 9.5 | 5 |  | pp | pp | death |
| BCAVF = brachiocepharic AVF, pp = primary patency maintained, RCAVF = radiocepharic AVF, sp = secondary patency maintained, TCC = tunneled cuffed catheter placement, UBAVF = ulnobasilic AVF, US = ultrasound, VP = venous pressure, | | | | | | | | | |
